# ESCRT Proteins Control the Dendritic Morphology of Developing and Mature Hippocampal Neurons

**DOI:** 10.1007/s12035-018-1418-9

**Published:** 2018-11-07

**Authors:** Marcelina Firkowska, Matylda Macias, Jacek Jaworski

**Affiliations:** 1 Laboratory of Molecular and Cellular Neurobiology, International Institute of Molecular and Cell Biology, Ks. Trojdena St. 4, 02-109 Warsaw, Poland; 2 Core Facility, International Institute of Molecular and Cell Biology, 02-109 Warsaw, Poland

**Keywords:** Neurons, Dendritic arbor, ESCRT, Vps25, mTOR

## Abstract

The proper shape of dendritic arbors of different types of neurons determines their proper communication within neuronal networks. The shape of dendritic arbors is acquired during a complex and multistep process called dendritogenesis. In most cases, once proper morphology is achieved, it remains stable throughout the lifespan, with the exception of rare events during which dendrites are abruptly pruned. The endosomal sorting complex required for transport (ESCRT) is multisubunit machinery that is involved in various cellular processes when membrane scission is needed. ESCRT subcomplexes regulate dendrite pruning in *Drosophila* neurons. However, the contribution of ESCRT components to the dendritogenesis of mammalian neurons and control of dendrite stability remains poorly defined. In the present study, we found that ESCRT-0, ESCRT-I, ESCRT-II, and ESCRT-III and Vps4 are required for proper dendrite morphology under basal culture conditions and for accelerated dendritogenesis in response to phosphoinositide 3-kinase (PI3K) activation. The knockdown of Vps28 (ESCRT-I) and Vps25 (ESCRT-II) resulted in downregulation of the activity of mechanistic/mammalian target of rapamycin complex 1. We also demonstrated that Vps28, Vps24, and Vps25 are required for dendrite stabilization in mature neurons.

**Electronic supplementary material:**

The online version of this article (10.1007/s12035-018-1418-9) contains supplementary material, which is available to authorized users.

## Introduction

Dendrites are a compartment of neurons that specialize in receiving and computing synaptic inputs from other cells within a given neuronal network. Some neurons possess only a single dendrite, whereas other neurons develop many dendrites and form very complex dendritic arbors. Both the genetic program and extracellular environment impact the final shape of dendritic arbors [1]. The formation of dendritic arbors, called dendritogenesis, is a complex molecular process that consists of individual dendrite growth, branching, and in some cases retraction (e.g., when dendrites meet other dendrites of the same cell). Once formed, dendritic arbors become less dynamic, but the majority of mature dendrites can be abruptly removed under certain conditions [2]. The molecular mechanism of mature dendrite stabilization is poorly understood, but it is known to only partially overlap with the mechanism that is responsible for dendritogenesis [2].

The endosomal sorting complex required for transport (ESCRT) is a multisubunit machinery that is mostly known for its role in generating multivesicular bodies (MVBs; i.e., membranous compartments that are critical for sorting endocytosed cargo for lysosomal degradation) [3, 4]. Nevertheless, the ESCRT as a whole complex or its subcomplexes are also important for the formation of exosomes and microvesicles, virus budding, abscission of the intercellular bridge during cytokinesis, nuclear envelope reformation, repair, quality control, and repair of the plasma membrane [5]. An additional role for ESCRT was described in neurons, in which it severs proximal dendrites during the dendrite pruning of dendritic arborization (*da*) neurons in *Drosophila* at the time of pupae metamorphosis [6, 7]. The ESCRT core consists of four major subcomplexes: ESCRT-0, ESCRT-I, ESCRT-II, and ESCRT-III. An additional protein that is needed for proper ESCRT function is VPS4, an adenosine triphosphatase, the activity of which allows for ESCRT-III recycling and force generation for membrane scission [8]. Aside from the major ESCRT components, it also consists of accessory proteins (e.g., Alix and His domain-containing protein tyrosine phosphatase [HD-PTP]), which may provide the additional or alternative recruitment of ESCRT modules to the membrane. In the canonical view of MVB formation, the sequential activity of all ESCRT components is required. However, the modular nature of the ESCRT and parallel interactions between subunits of each subcomplex and accessory proteins allows several exceptions of this canonical order of events. For example, ESCRT-III can be recruited to the membrane by either ESCRT-I or Alix, bypassing ESCRT-II [5, 9]. Thus, some functions of the ESCRT may not require the participation of all of its subcomplexes. Conversely, some ESCRT subcomplexes have non-canonical functions beyond the ESCRT. For example, in *Drosophila* oocytes, ESCRT-II binds Staufen, an RNA binding protein, and regulates the polarized distribution of *bicoid* RNA [10]. Konopacki et al. [11] suggested that ESCRT-II is exclusively required for the coupling of endocytosis with local protein synthesis during *Xenopus* retinal ganglion cell axon navigation. Whether a similar mechanism operates during dendritogenesis remains unknown.

To date, most data that link ESCRT proteins with dendritic arbor growth or remodeling have come from studies of *Drosophila* metamorphosis. During this process, *da* neurons need to develop fully mature dendritic arbors in less than 20 h, which then undergo massive pruning immediately before the pupae becomes a fly. During *da* neuron development, Snf7/Shrub (ESCRT-III) is needed for the removal of supernumerary dendrites [6, 12]. The knockdown or knockout of components of ESCRT-I or ESCRT-III caused a lack of dendrite pruning in mature *da* neurons [6, 7]. This effect was attributed to the lack of downregulation of the cell adhesion protein Neuroglian, which supports dendrite stabilization [7]. In contrast to *Drosophila*, Snf7 or CHMB2B knockdown or *Alix* knockout in mammalian cells leads to the simplification of dendritic arbor morphology [13–16]. These observations suggest that the contribution of ESCRTs to dendrite pruning may not be conserved between *Drosophila* and mammalian neurons. The participation of ESCRTs in dendritic arbor growth in mammals remains largely unknown.

In the present study, we performed a short-hairpin RNA (shRNA)-based mini screen to elucidate the role of individual ESCRT proteins in the acquisition of proper dendritic arbor morphology in developing hippocampal neurons. We found that all ESCRT subcomplexes (i.e., ESCRT-0, ESCRT-I, ESCRT-II, and ESCRT-III) and Vps4 were required for this process under basal culture conditions. We also found that ESCRT-I, ESCRT-II, and ESCRT-III and Vps4 are needed for phosphoinositide 3-kinase (PI3K)-induced, mechanistic/mammalian target of rapamycin (mTOR)-dependent dendritogenesis. The knockdown of Vps28 (ESCRT-I) and Vps25 (ESCRT-II) resulted in downregulation of the activity of mTOR complex 1 (mTORC1). Based on the involvement of ESCRT modules in dendrite pruning in *Drosophila*, we also tested their importance for dendritic arbor morphology in mature hippocampal neurons and showed that the removal of Vps28, Vps25, and Vps24 (ESCRT-III) negatively affected the number of dendrites.

## Methods

### Antibodies and Drugs

The following antibodies were obtained from commercial sources: rabbit anti-green fluorescent protein (GFP; catalog no. 598, Medical and Biological Laboratories, Woburn, MA, USA), rabbit anti-S6 ribosomal protein (catalog no. 2217, Cell Signaling Technology, Danvers, MA, USA), rabbit anti-phospho-S6 (Ser-240/244, catalog no. 2215, Cell Signaling Technology), rabbit anti-phospho-Akt (Ser-473, catalog no. 4060, Cell Signaling Technology), mouse anti-Akt (catalog no. 2920, Cell Signaling Technology), mouse anti-β-galactosidase (catalog no. Z3781, Promega, Madison, WI, USA), rabbit anti-β-galactosidase (catalog no. 559762, MP Biomedicals, Santa Ana, CA, USA), mouse anti-monoubiquitinylated and polyubiquitinylated conjugates (clone FK; catalog no. BML-PW8810, Enzo Life Sciences, Farmingdale, NY, USA), and mouse anti-tubulin (catalog no. T5168, Sigma-Aldrich, St. Louis, MO, USA). Anti-mouse and anti-rabbit secondary antibodies conjugated to Alexa Fluor 488 or Alexa Fluor 647 were obtained from Invitrogen (Carlsbad, CA, USA). IRDye-conjugated secondary donkey anti-mouse IRDye 680RD antibody (catalog no. 926-68072) and donkey anti-rabbit IRDye 800CW antibody (catalog no. 926-32213) were obtained from LI-COR Biosciences (Lincoln, NE, USA). Insulin was obtained from Sigma-Aldrich (St. Louis, MO, USA).

### DNA Constructs

The following mammalian expression plasmids have been previously described: pSuper [17], pEFα-βGal [18], pβ-actin-GFP (encoding enhanced GFP [EGFP] under control of the β-actin promoter) [19], p110-CAAX [19], pSuper-mTOR7513 [19], pCMV-VSV-G [20], RSV-Rev [21], and pMDLg/pRRE [21]. The sequences that encode the shRNAs that were used in the ESCRT screen were cloned into pSuper and are listed in Table 1. Lentiviral vectors that expressed the Vps28#1, Vps25#1, and Vps24#1 shRNAs were generated by subcloning shRNA-encoding sequences from pSuper using SmaI/XhoI into analogous sites of pUltra-Chilli vector (a gift from Malcolm Moore; Addgene plasmid no. 48687).

**Table 1:** shRNA targeting sequences used in the study

Complex	shRNA name	NCBI reference sequence	shRNA sequences
ESCRT-0	shHRS	NM_019387.2	5′-CTAGCCATTTCCTCCCATT-3′ 5′-AGGATGAGACAGAAGTCAA-3′ 5′-AGCCATACAACATGCAGAA-3′
ESCRT-I	shVps28#1	NM_001130492.1	5′-GAAGTAAAGTTGTACAAGA-3′ 5′-CTATGGAGCGGATCAAAGA-3′ 5′-CCTTCAACCGCTTCCTACA-3′
ESCRT-II	shVps22	NM_001007804.1	5′-GAGCTCAATATGGATCACA-3′ 5′-GCGTCCAGATTATTGAAGT-3′ 5′-GGGACTTCTATTATGAACT-3′
	shVps25	NM_001173451.1	5′-GTGGCCAGAATAACTCTGT-3′ 5′-CTCGAGTGGTTGGATAAGA-3′ 5′-GGCCAGAATAACTCTGTGT-3′
	shVps36	NM_001106092.2	5′-GTGGTGTCATGGTAATTGA-3′ 5′-CGATCGCTAATAAGATTAA-3′ 5′-GAGGTGTACTGTTTAGTGA-3′
ESCRT-III	shVps24	NM_172331.1	5′-GAAGGCTGGAATCATAGAA-3′ 5′-GAGGATACGTTTGAAAGCA-3′ 5′-GCTCTATGCATCCAAAGCA-3′
	shVps20	NM_001105856.1	5′-GCATTGAGTTCACGCAGAT-3′ 5′-GTCTGAATAAGATGCACCA-3′ 5′-GGGAACGAATGTCTGAATA-3′
	shVps2B	XM_343995.7	5′-CCACAGAAGACACTACAAA-3′ 5′-CGTGAATCAAGTTCTTGAT-3′
	shSnf7	NM_001017466.2	5′-GATGACTTGATGCAAGACA-3′ 5′-CTGAGGTCTTACGGAACAT-3′ 5′-CATGAAAGCTGTTCATGAA-3′
	shDid2	NM_001083313.1	5′-CCTCATCGTTCAGATTGCT-3′ 5′-GATACCCTGTTCCAGTTGA-3′ 5′-CTGTTCCAGTTGAAGTTCA-3′
	shVps60	NM_001025410.1	5′-CCACGGTTGATGCAATGAA-3′ 5′-GCAATGAAATTGGGAGTAA-3′ 5′-GCTAATTACACCATCCAGT-3′
AAA ATPase	shVps4A#1	NM_145678.1	5′-CCCTAGTGTTATGATTGAT-3′ 5′-CCCAGATGTTTCGGTTACA-3′ 5′-CAGAGTTCTTGGTCCAAAT-3′

### Neuronal Culture and Transfection

The animals that were used to obtain neurons for further experiments were sacrificed according to protocols that were approved by the First Ethical Committee in Warsaw (no. 400/2012), which complied with European Community Council Directive 2010/63/EU. Primary hippocampal and cortical cultures were prepared from embryonic day 19 (E19) rat brains and transfected on day in vitro (DIV) 7, DIV18 (hippocampal), or DIV0 (cortical) using Lipofectamine 2000 (Invitrogen) or the Amaxa nucleofection procedure (Lonza, Switzerland), respectively, as described previously [22]. For the shRNA ESCRT screen, the DIV7 neurons were transfected with a pool of plasmids that encoded three shRNAs (with the exception of shRNAs against Vps2B) that targeted a given gene and β-actin-GFP. For PI3K-induced dendritic growth, DIV7 neurons were cotransfected using a pool of plasmids that encoded three different shRNAs that targeted the gene or with plasmids that encoded individual, selected shRNA and p110-CAAX or β-Gal together with β-actin-GFP. Mature neurons (DIV18) were transfected with pSuper that encoded individual shRNA and a GFP-encoding vector.

### Lentiviral Vector Production, Transduction, and Drug Treatment

Lentiviral vectors were produced in HEK293T cells using pUltra-Chilli and pUltra-Chilli-shRNA lentiviral vectors and packaging plasmids (pCMV-VSV-G, pRSV-Rev, and pMDLg-pRRE) as described previously [23]. Two days after transfection, the virus-containing medium was collected and passed through a 0.45-μm filter to remove cellular debris. Cortical neurons were transduced with lentivirus on DIV3 for 7 days to ensure efficient shRNA-mediated knockdown of the protein of interest. Afterward, insulin (400 nM) was added to the cells for 20 min. The drug application was preceded by overnight starvation in Neurobasal medium (Thermo Fisher Scientific, Waltham, MA, USA) supplemented with 1% penicillin-streptomycin (Sigma-Aldrich) and 0.5 mM glutamine (Thermo Fisher Scientific) but without B27 supplementation. The cells were collected and processed for immunoblotting as described below.

### Western Blot

Protein concentrations were first measured using the Pierce BCA Protein Assay Kit (Thermo Fisher Scientific). The proteins were then denatured in Laemmli buffer. The samples were separated by sodium dodecyl sulfate-polyacrylamide gel electrophoresis and transferred to nitrocellulose membranes. The membranes were blocked in 5% non-fat milk in TBS buffer. For quantitative analysis, we used Western blot signal detection with the Infrared Odyssey Imaging System (LI-COR Biosciences). Membranes with transferred proteins, after incubation with primary antibodies, were washed three times with TBS-T (10 mM Tris [pH 8.0], 150 mM NaCl, and 0.1% Tween-20) and incubated with appropriate IRDye680 and IRDye800 secondary antibodies in TBS-T with 5% non-fat milk for 1 h. The blots were then washed with TBS-T and deionized water. Dried membranes were used for fluorescence signal acquisition using the Infrared Odyssey Imaging System. The results were quantified using Image Studio Lite (LI-COR Biosystems). The signal intensity from phosphorylated and total protein was first normalized to respective tubulin levels, and then, the phosphorylated/total protein ratio was calculated.

### Immunofluorescence Staining

For the immunofluorescence staining of overexpressed proteins, neurons were fixed with 4% paraformaldehyde that contained 4% sucrose in phosphate-buffered saline (PBS; 137 mM NaCl, 2.7 mM KCl, 8 mM Na_2_HPO_4_, and 1.4 mM KH_2_PO_4_, pH 7.4) for 10 min at room temperature and stained for β-Gal or GFP diluted 1:1000 in GDB buffer (30 mM phosphate buffer [pH 7.4], 0.2% gelatin, 0.5% Triton X-100, and 450 mM NaCl). The next day, the coverslips were incubated with Alexa Fluor 647- or Alexa Fluor 488-conjugated antibody diluted 1:200 in GDB and mounted using Prolong Gold (Invitrogen). For the immunofluorescence staining of monoubiquitinylated and polyubiquitinylated conjugates, cell fixation was followed by 4 min of cell permeabilization with 0.1% Triton X-100 in PBS. The cells were then incubated with blocking solution (3% bovine serum albumin in PBS) for 30 min. Primary antibodies against monoubiquitinylated and polyubiquitinylated conjugates (FK) and against β-Gal were diluted 1:200 and 1:2000, respectively, in the blocking solution and added to the cells overnight. The cells were then washed three times with PBS and incubated with appropriate secondary antibodies conjugated with Alex Fluor.

### Electron Microscopy

To obtain sections for electron microscopy, rat cortical neurons were grown in vitro on Thermanox coverslips (Thermo Fisher Scientific). On DIV10, the cells were fixed with 2.5% glutaraldehyde in PBS for 120 min and washed twice with PBS. Afterward, the cells were incubated with 1% osmium tetraoxide for 1 h, washed three times with deionized water and then in increasing concentrations of ethanol, and then incubated with 1% uranyl acetate for 40 min. The samples were then dehydrated in ethanol, followed by pure propylene oxide. The samples were then embedded in EPON resin (Sigma-Aldrich), cut into ultrathin sections (60 nm) using a Leica EM UC7 microtome, and mounted on copper grids (Leica Microsystems, Wetzlar, Germany). Photomicrographs were taken using a Tecnai T12 BioTwin electron microscope (FEI, Hillsboro, OR, USA) at ×18,500 magnification.

### RNA Isolation and Reverse-Transcription Quantitative Polymerase Chain Reaction

RNA from cultured cortical neurons was isolated with the RNeasy Protect Minikit (Qiagen, Venlo, The Netherlands). cDNA was prepared with High Capacity RNA-to-cDNA Master Mix (Applied Biosystems, Foster City, CA, USA) according to the manufacturer’s protocol. Quantitative polymerase chain reaction (qPCR) was performed using a 7900HT real-time PCR system and TaqMan gene expression assays (Applied Biosystems) with the following TaqMan rat probes: GAPDH (Rn99999916_s1), tubulin (Rn01532518_g1), Hrs (Rn00693812_m1), Vps28 (Rn01768029_m1), Vps25 (Rn01751306_m1), Vps36 (Rn01478763_m1), Vps22 (Rn01460691_m1), Vps24 (Rn00597295_m1), Vps20 (Rn01452925_g1), Vps2B (Rn01402241_m1), Snf7 (Rn01461785_mH), Did2 (Rn01432784_m1), Vps60 (Rn01764717_m1), and Vps4A (Rn00595538_m1). The results were analyzed using the comparative Ct method for relative quantification. SDS 2.4 and RQ Manager 1.2.1 software programs were used for data acquisition and preliminary analysis.

### Microscopy and Image Analysis

Images of fluorescently labeled in vitro cultured neurons were acquired using a Zeiss LSM5 Exciter confocal microscope (×40 oil objective), Zeiss LSM710 NLO (×20 air objective), or Zeiss LSM 800 (×40 oil objective, 0.9 digital zoom) at 1024 × 1024 pixel resolution. *Z*-stacks of the images were averaged twice per line, and the settings were kept constant for all of the scans. The *Z*-stacks were converted to single images using a maximum intensity projection. Morphometric analysis and quantification were performed using MetaMorph image analysis software (Universal Imaging, Downingtown, PA, USA) to manually count the total number of dendritic tips (TNDT). Counts of the number of FK2-positive particles were made in the defined region of interest (cell body) selected based on the β-Gal immunofluorescence. The number of particles larger than 2 pixels was analyzed. Laser parameters for scanning and threshold for image analysis were kept constant for all four experiments.

### Statistical Analysis

The data were obtained from at least three independent batches of cells. The exact numbers of cells are provided in the respective figure legends. The statistical analyses were performed using Prism software (GraphPad, San Diego, CA, USA). The data were analyzed using the Kruskal-Wallis test followed by Dunn’s post hoc test, one-sample *t* test, or correlation test, depending on the type of data analyzed.

## Results

### ESCRT Complex Proteins Are Needed for Dendritic Growth Under Basal Conditions

**Fig. 1:**
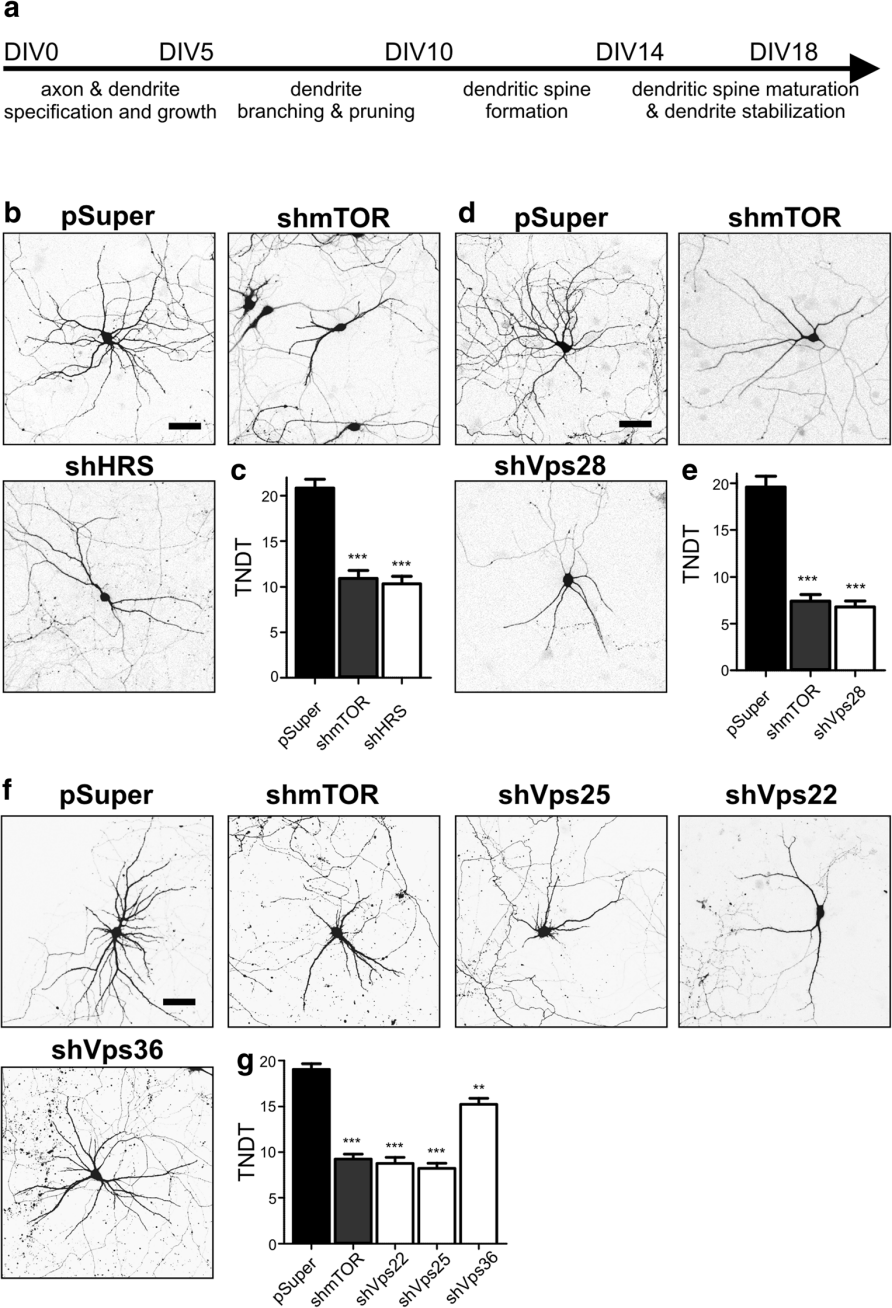
Knockdown of Hrs (representing ESCRT-0), Vps28 (representing ESCRT-I), and ESCRT-II complex proteins simplifies dendritic arbors of hippocampal neurons. **a** Developmental timeline of neurons cultured in vitro. **b** Representative images of neurons that were transfected on DIV7 for 4 days with pSuper, pSuper-shmTOR7513, or a pool of pSuper plasmids that encoded three different Hrs shRNAs. For the visualization of neuronal morphology, the cells were cotransfected with a GFP-encoding plasmid. Scale bar = 40 μm. **c** Total number of dendritic tips (TNDT) of cells that were transfected as in **b**. Cell images were obtained from three independent experiments. Number of analyzed cells: pSuper (*n* = 50), shmTOR (*n* = 58), shHRS (*n* = 46). Error bars indicate the SEM. ***p < 0.001, compared with pSuper (Kruskal-Wallis test followed by Dunn’s post hoc test). **d** Representative images of neurons that were transfected on DIV7 for 4 days with pSuper, pSuper-shmTOR7513, or a pool of pSuper plasmids that encoded three different Vps28 shRNAs. For the visualization of neuronal morphology, the cells were cotransfected with a GFP-encoding plasmid. Scale bar = 40 μm. **e** TNDT of cells that were transfected as in **d**. Cell images were obtained from three independent experiments. Number of analyzed cells: pSuper (*n* = 55), shmTOR (*n* = 47), shVps28 (*n* = 48). Error bars indicate the SEM. ***p < 0.001, compared with pSuper (Kruskal-Wallis test followed by Dunn’s post hoc test). **f** Representative images of neurons that were transfected on DIV7 for 4 days with pSuper, pSuper-shmTOR7513, or a pool of pSuper plasmids that encoded three different shRNAs against Vps22, Vps25, or Vps36. For the visualization of neuronal morphology, the cells were cotransfected with a GFP-encoding plasmid. Scale bar = 40 μm. **g** TNDT of cells that were transfected as in **f**. Cell images were obtained from three independent experiments. Number of analyzed cells: pSuper (*n* = 66), shmTOR (*n* = 62), shVps22 (*n* = 51), shVps25 (*n* = 45), shVps36 (*n* = 60). Error bars indicate the SEM. ***p < 0.001, compared with pSuper (Kruskal-Wallis test followed by Dunn’s post hoc test)

**Fig. 2:**
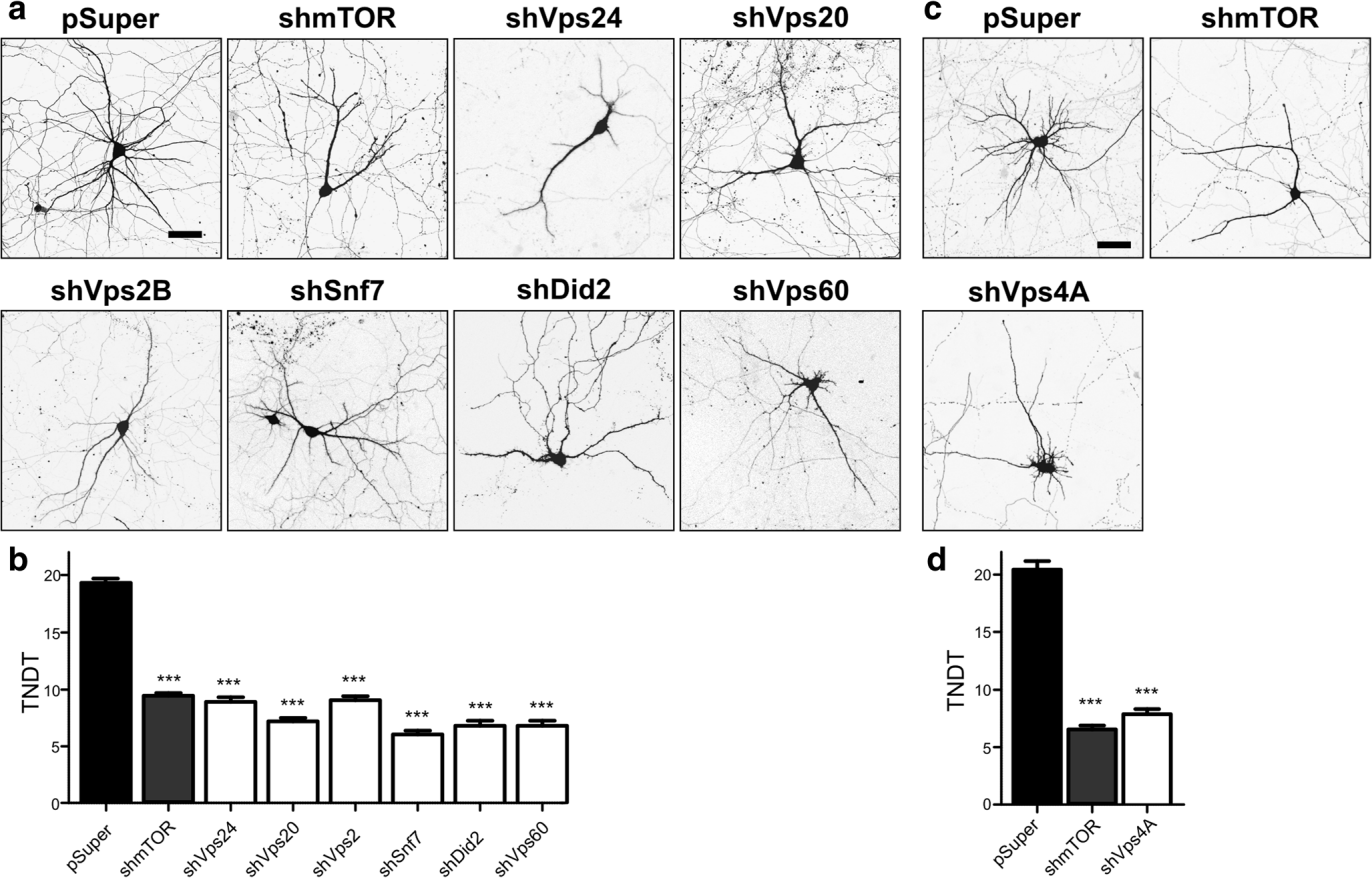
Knockdown of ESCRT-III complex proteins and Vps4A simplifies dendritic arbors of hippocampal neurons. **a** Representative images of neurons that were transfected on DIV7 for 4 days with pSuper, pSuper-shmTOR7513, or a pool of pSuper plasmids that encoded at least two different shRNAs against Vps24, Vps20, Vps2, Snf7, Did2, or Vps60. For the visualization of neuronal morphology, the cells were cotransfected with a GFP-encoding plasmid. Scale bar = 40 μm. **b** Total number of dendritic tips (TNDT) of cells that were transfected as in **a**. Cell images were obtained from three independent experiments. Number of analyzed cells: pSuper (*n* = 60), shmTOR (*n* = 58), shVps24 (*n* = 51), shVps20 (*n* = 49), shVps2 (*n* = 53), shSnf7 (*n* = 38), shDid2 (*n* = 54), shVps60 (*n* = 53). Error bars indicate the SEM. ***p < 0.001, compared with pSuper (Kruskal-Wallis test followed by Dunn’s post hoc test). **c** Representative images of neurons that were transfected on DIV7 for 4 days with pSuper, pSuper-shmTOR7513, or a pool of pSuper plasmids that encoded three different Vps4A shRNA. For the visualization of neuronal morphology, the cells were cotransfected with a GFP-encoding plasmid. Scale bar = 40 μm. **d** TNDT of cells that were transfected as in **c**. Cell images were obtained from three independent experiments. Number of analyzed cells: pSuper (*n* = 57), shmTOR (*n* = 59), shVps4A (*n* = 60). Error bars indicate the SEM. ***p < 0.001, compared with pSuper (Kruskal-Wallis test followed by Dunn’s post hoc test)

Previous research on *Drosophila* neurons showed that several components of ESCRT machinery are needed to prune dendritic arbors of *da* neurons during metamorphosis [6, 7]. Studies of ESCRT-III in mammalian neurons suggested that ESCRT-III is needed for the maturation of dendritic spines (structural equivalents of excitatory synapses) and mature dendrite stabilization [13, 15, 16]. However, a comprehensive analysis of the contribution of the ESCRT complex to dendrite development has not yet been performed. Therefore, we designed a mini-shRNA library in a pSuper vector that targeted selected subunits of rat ESCRT-0, ESCRT-I, ESCRT-II, and ESCRT-III and Vps4 (Table 1). When nucleofected to DIV0 cortical neurons for 3 days as mixtures of pSuper plasmids that encoded three different shRNAs per targeted mRNA (with the exception of Vps2B for which only two shRNAs could be designed), these shRNAs effectively downregulated the respective mRNA levels (Supplemental Fig. S1A; Online Resource 1). After confirming the efficacy of our shRNAs against their targets, we performed a mini-shRNA screen in developing hippocampal neurons that were cultured in vitro. Neurons that are cultured in vitro go through identical developmental stages as in vivo neurons. During the first 3 days, polarization, axon formation, and dendritic growth occur. Intense dendritogenesis, including dendrite formation and retraction, occurs from DIV5 to DIV10. At the end of this period, the formation of dendritic spines begins. After DIV14, dendritic spines start to mature, and the dendritic tree finally stabilizes (Fig. 1a). Thus, to study the effects of ESCRT subunit knockdown on dendritogenesis, DIV7 neurons were transfected with a mixture of pSuper that encoded three different shRNAs that targeted each mRNA together with a pβ-actin-GFP-encoding EGFP plasmid under control of the β-actin promoter (for the visualization of transfected cell morphology). As a control, neurons were transfected with pSuper (negative control) or pSuper that encoded previously validated shRNA against mTOR (positive control for dendritic arbor simplification [19]). Our previous studies showed that for the analysis of dendritic arbor complexity, the effects of pSuper and non-targeting or scrambled shRNA transfection were not significantly different [24, 25]. Four days after transfection, the cells were fixed. Confocal images were acquired, and the TNDT was quantified. As shown in Fig. 1b, c, the knockdown of Hrs (ESCRT-0) resulted in 50% fewer dendrites, similar to mTOR knockdown, compared with pSuper-transfected controls. Similar results were obtained for the knockdown of Vps28 (ESCRT-I; Fig. 1d, e), Vps22, Vps25, Vps36 (ESCRT-II; Fig. 1f, g), Vps24, Vps20, Vps2B, Snf7, Did2, Vps60 (ESCRT-III; Fig. 2a, b), and Vps4A (Fig. 2c, d). Thus, all ESCRT machinery modules were needed for the proper dendritic arbor morphology of developing neurons.

### ESCRT-I, ESCRT-II, and ESCRT-III Are Needed for the Induction of Dendritic Growth

**Fig. 3:**
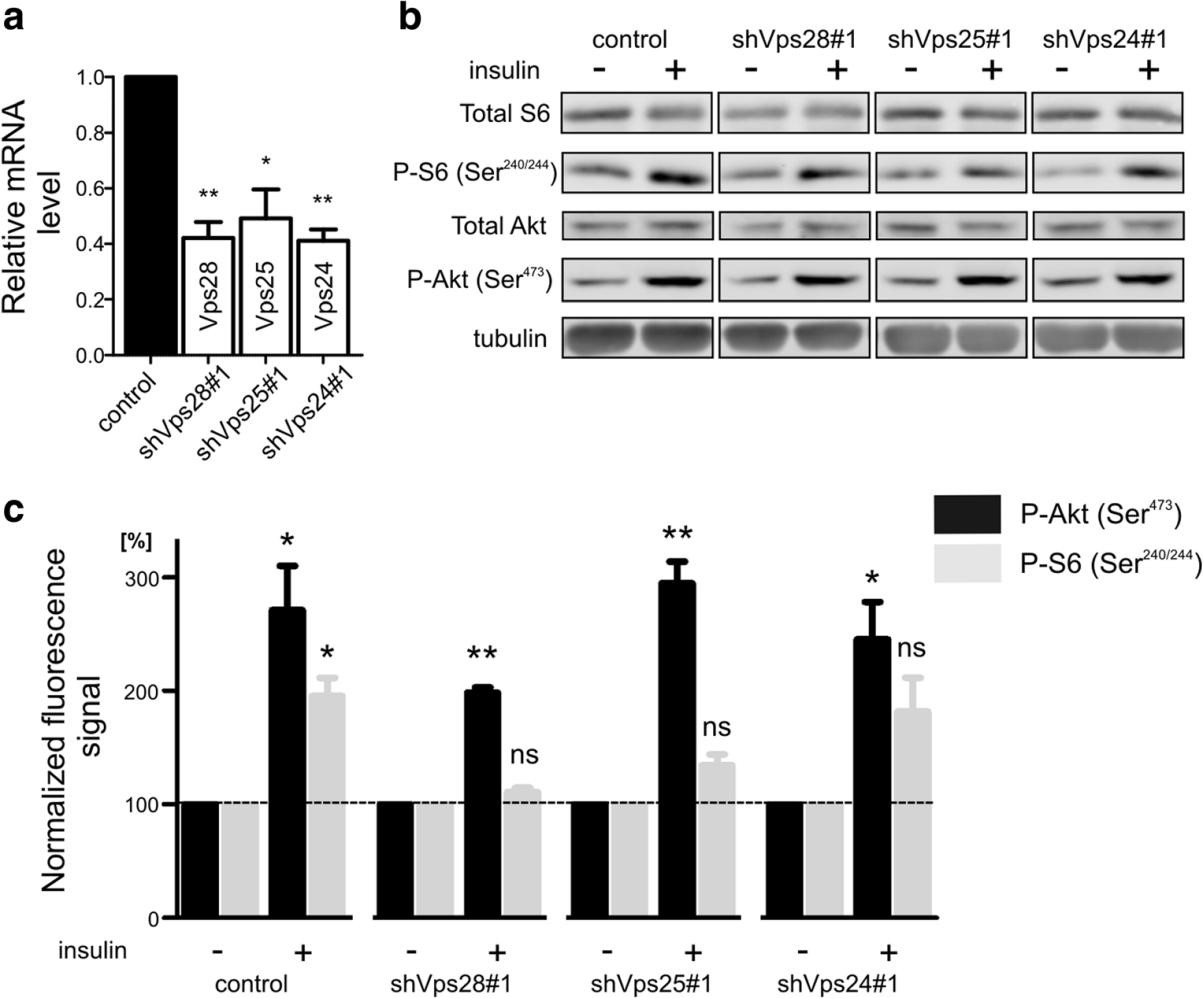
Knockdown of ESCRT-I, ESCRT-II, and ESCRT-III affects mTOR signaling. **a** The results of the qRT-PCR-based analysis of the indicated gene expression in cortical neurons that were transduced on DIV3 with control lentiviral vector (based on pUltra-Chilli plasmid) or lentiviral vectors that expressed Vps28#1, Vps25#1, and Vps24#1 shRNAs for 7 days. GAPDH mRNA was used as a reference. The plot represents mean 2(− ΔΔCt) values ± SEM. The data were derived from three independent experiments. *p < 0.05, **p < 0.01, ***p < 0.001 (one-sample *t* test). **b** Representative Western blots that show S6, phospho-S6 (Ser240/244), Akt, and phospho-Akt (Ser473) levels in cortical neurons that were treated with insulin (400 mM, 20 min) upon the knockdown of Vps28, Vps25, and Vps24 as in **a**. Tubulin is shown as a loading control. **c** Results of Western blot analysis of normalized P-S6 and P-Akt levels in protein lysates from neurons that were transduced as in **a**. The signal was normalized to control levels (illustrated as a line). Error bars indicate the SEM. The data were derived from three independent experiments. *p < 0.05, **p < 0.01, compared with control value = 100 (one-sample *t* test); ns not significant

The induction of dendritogenesis by the overexpression of a constitutively active PI3K mutant, trophic factors, or insulin requires mTORC1 and mTORC2 [24–26]. Data from yeast suggest that ESCRTs are functionally linked to mTOR signaling [27]. Therefore, we investigated whether ESCRTs in developing neurons cultured in vitro control insulin-induced mTORC1 and mTORC2 signaling. DIV3 neurons were transduced with lentiviral vectors that encoded shRNAs that targeted selected representatives of ESCRT-I, ESCRT-II, or ESCRT-III for 7 days to ensure efficient shRNA-mediated knockdown of the protein of interest during the period of intensive dendritogenesis and treated with insulin for 20 min. The activation of mTORC1 and mTORC2 was analyzed by the Western blot assessment of rpS6 (S240/244) and Akt (S473) phosphorylation, respectively. Upon transduction, the levels of mRNA that encoded respective ESCRT subunits decreased substantially (Fig. 3a). The knockdown of Vps28 and Vps25 proteins affected P-S6 levels in insulin-treated cells but not in control cells (Fig. 3b, c). The knockdown of Vps24 also prevented mTORC1 stimulation by insulin, but the effect was not statistically significant. At the same time, P-Akt levels remained largely unaffected.

**Fig. 4:**
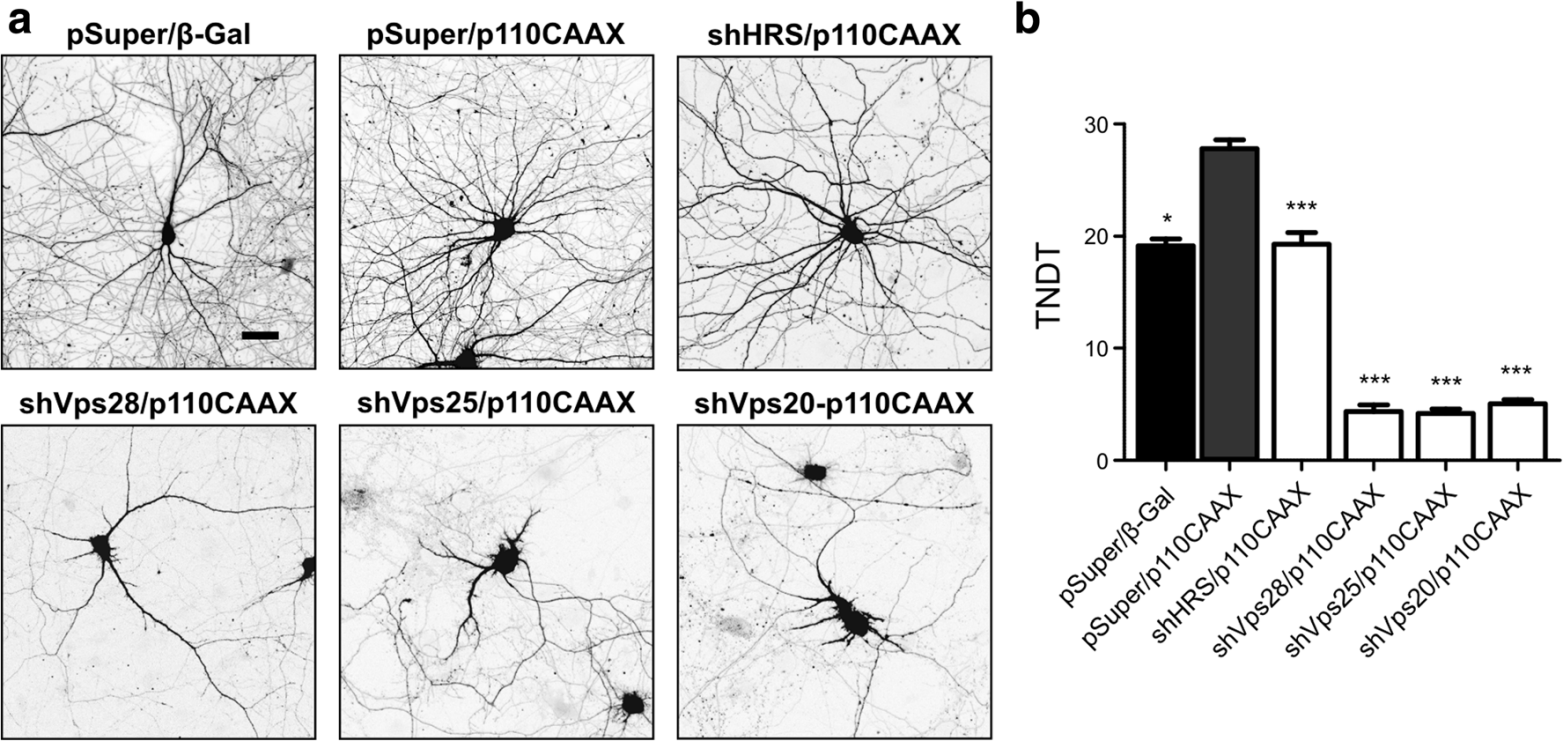
Knockdown of ESCRT-I, ESCRT-II, and ESCRT-III effectively inhibits PI3K-stimulated dendritogenesis. **a** Representative images of neurons that were transfected on DIV7 for 5 days with p110-CAAX or β-Gal (negative control) plasmids and pSuper or pSuper plasmids that encoded three different shRNAs against HRS, Vps28, Vps25, and Vps20. For the visualization of neuronal morphology, the cells were cotransfected with a GFP-encoding plasmid. Scale bar = 40 μm. **b** Total number of dendritic tips (TNDT) of cells that were transfected as in **a**. Cell images were obtained from three independent experiments. Number of analyzed cells: pSuper/β-Gal (*n* = 65), pSuper/p110CAAX (*n* = 63), shHRS mix/p110CAAX (*n* = 56), shVps28 mix/p110CAAX (*n* = 46), shVps25mix/p110CAAX (*n* = 47), shVps20-p110CAAX (*n* = 60). Error bars indicate the SEM. ***p < 0.001, *p < 0.05, compared with pSuper/p110-CAAX (Kruskal-Wallis test followed by Dunn’s post hoc test)

Knowing that ESCRT proteins are needed for the induction of mTOR signaling, we next investigated whether ESCRT subcomplexes are needed for neurodevelopment that is accelerated by the genetic overactivation of PI3K. DIV7 neurons were transfected with pSuper or a mixture of three pSuper plasmids that targeted selected representatives of ESCRT-0, ESCRT-I, ESCRT-II, or ESCRT-III together with p110-CAAX that encoded constitutively active PI3K (Fig. 4). As a control, neurons were transfected with pSuper and pEFα-βGal. Analogously to the experiments that are described above, a GFP-encoding vector was cotransfected for the visualization of transfected cell morphology. Five days post-transfection, the neurons were fixed, and TNDT was calculated. As previously reported (e.g., [19, 24]), the overexpression of p110-CAAX resulted in a significant increase in TNDT. The knockdown of each ESCRT subcomplex effectively prevented such accelerated dendritogenesis, and ESCRT-0 knockdown had the mildest effect (Fig. 4). Similar effects were observed with the transfection of pSuper plasmids that encoded individual shRNAs that targeted selected ESCRT proteins instead of plasmid pools (Supplementary Figs. S1B and S2; Online Resource 1). Thus, the presence of ESCRT-I, ESCRT-II, and ESCRT-III is required for mTOR activation and PI3K-induced dendritic growth in developing neurons.

### Knockdown of ESCRT-I, ESCRT-II, and ESCRT-III Results in the Accumulation of Ubiquitinylated Proteins in Neurons

**Fig. 5:**
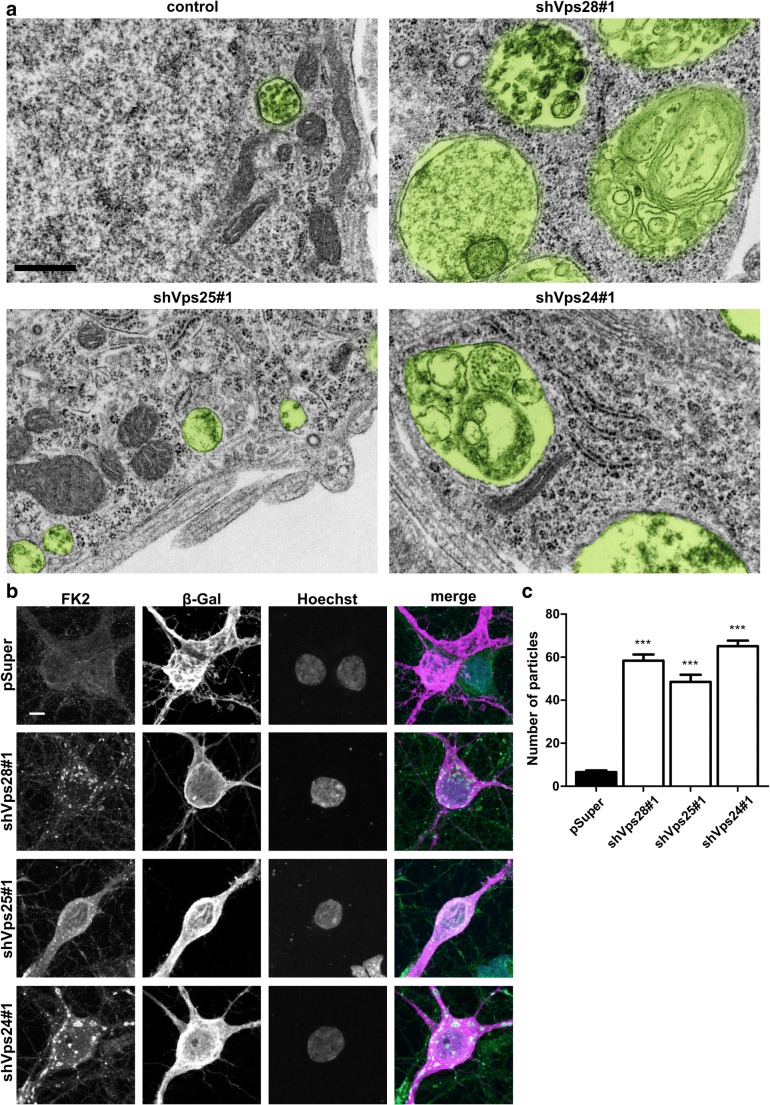
Knockdown of ESCRT-I, ESCRT-II, and ESCRT-III results in the accumulation of ubiquitinylated proteins in developing neurons. **a** Representative electron microscopy images of cortical neurons transduced on DIV3 for 7 days with pChilli, pChilli-shVps28#1, pChilli-shVps25#1, and pChilli-shVps24#1. MVBs are highlighted in green. Scale bar = 500 nm. **b** Representative images of hippocampal neurons transfected on DIV7 for 4 days with pSuper, pSuper-shVps28#1, pSuper-shVps25#1, and pSuper-shVps24#1. For the visualization of neuronal morphology, the cells were cotransfected with a β-Gal-encoding plasmid. Scale bar = 10 μm. **c** Average number of FK antibody-positive particles > 2 pixels in the cell body of neurons transfected as in **b**. Cell images were obtained from four independent experiments. Number of analyzed cells: pSuper (*n* = 89), shVps28#1 (*n* = 76), shVps25#1 (*n* = 70), shVps24#1 (*n* = 83). Error bars indicate the SEM. ***p < 0.001, compared with pSuper (Kruskal-Wallis test followed by Dunn’s post hoc test)

The data above indicate that the knockdown of all ESCRT proteins resulted in the simplification of developing dendritic arbors. To confirm that this phenotype is attributed to the canonical function of ESCRTs (i.e., MVB formation and steering ubiquitinylated proteins for lysosomal degradation), we checked the ultrastructure and accumulation of ubiquitinylated proteins in neurons upon the knockdown of selected ESCRT-I, ESCRT-II, and ESCRT-III components. Neurons were transduced with lentiviral vectors that encoded shRNAs against Vps28, Vps25, or Vps24 as described above and analyzed on DIV10 for changes in ultrastructure using electron microscopy. Cells with Vps28 and Vps24 knockdown exhibited apparent changes of MVB morphology (Fig. 5a) that resembled those that were previously reported for non-neuronal cells with ESCRT-I and ESCRT-III dysfunction. Vps25 knockdown did not cause such noticeable differences compared with controls.

To further confirm the disturbance of ESCRT function, we took advantage of the fact that the inhibition of ESCRT subunits leads to the accumulation of ubiquitinated proteins [28]. Thus, we immunofluorescently labeled control neurons or neurons upon Vps28, Vps25, and Vps24 knockdown 3 days after transfection (DIV7 + 3) with FK antibody, which detects monoubiquitinylated and polyubiquitinylated conjugates, and quantified the number of aggregates that formed per cell. Very few aggregates were detected in pSuper-transfected cells, whereas the transfection of all tested shRNAs resulted in a significant increase in their formation (Fig. 5b, c). However, in the case of Vps25 knockdown, such FK particles were substantially smaller, which corresponded well with our ultrastructural observations. Thus, we concluded that the knockdown of ESCRT-I, ESCRT-II, and ESCRT-III in developing neurons leads to MVB dysfunction.

### ESCRT-I, ESCRT-II, and ESCRT-III Are Needed for the Stability of Dendritic Arbors of Mature Hippocampal Neurons

**Fig. 6:**
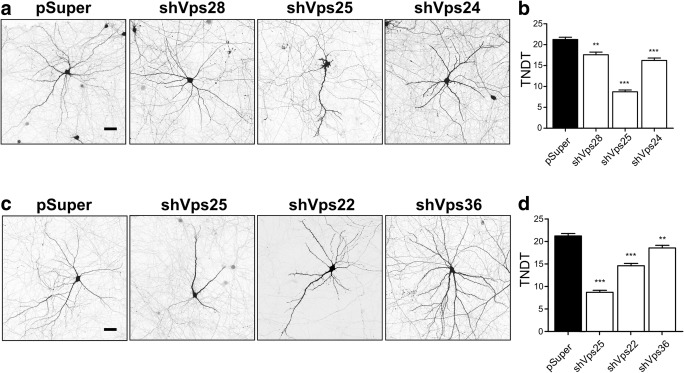
Knockdown of Vps28, Vps25, and Vps24 in mature neurons simplifies dendritic tree morphology. **a** Representative images of neurons that were transfected on DIV18 for 4 days with pSuper, pSuper-shVps28#1, pSuper-shVps25#1, or pSuper-shVps24#1. For the visualization of neuronal morphology, the cells were cotransfected with a GFP-encoding plasmid. Scale bar = 40 μm. **b** Total number of dendritic tips (TNDT) of cells that were transfected as in **a**. Cell images were obtained from four independent experiments. Number of analyzed cells: pSuper (*n* = 80), shVps28 (*n* = 78), shVps24 (*n* = 80), shVps25 (*n* = 81). Error bars indicate the SEM. ***p < 0.001, **p < 0.01, compared with pSuper (Kruskal-Wallis test followed by Dunn’s post hoc test). **c** Representative images of neurons that were transfected on DIV18 for 4 days with pSuper, pSuper-shVps25#1, shVps22#1, or shVps36#3. For the visualization of neuronal morphology, the cells were cotransfected with a GFP-encoding plasmid. Scale bar = 40 μm. **d** TNDT of cells that were transfected as in **c**. Cell images were obtained from four independent experiments. Number of analyzed cells: pSuper (*n* = 80), shVps25#1 (*n* = 81), shVps22#1 (*n* = 75), shVps36#3 (*n* = 80). Error bars indicate the SEM. ***p < 0.001, compared with pSuper (Kruskal-Wallis test followed by Dunn’s post hoc test); ns not significant

The aforementioned findings suggest that all ESCRT modules are needed for dendritic growth in developing neurons. However, studies by Lee et al. [13] and Goldberg and coworkers [15, 16] suggested that ESCRT-III might also be involved in the proper stabilization of mature dendritic arbors of cortical and hippocampal neurons. Therefore, we investigated whether other ESCRT modules in mature hippocampal neurons are also needed for this process. Dendritic arbors of hippocampal neurons in vitro become stable during the third week in culture. Thus, DIV18 neurons were transfected for 4 days with pSuper or pSuper that encoded shRNAs that targeted selected representatives of ESCRT-I, ESCRT-II, or ESCRT-III and GFP-encoding vector. The overexpression of all three shRNAs resulted in a reduction of TNDT, but only the transfection of Vps25 shRNA caused an effect that was comparable to the effect in developing neurons (i.e., ~50% reduction; Fig. 6a, b). Accordingly, we further investigated whether the knockdown of Vps22 and Vps36, two other subunits of ESCRT-II, has detrimental effects that are similar to Vps25 knockdown on the stability of mature dendritic arbors of hippocampal neurons. However, the knockdown of either Vps22 or Vps36 did not result in such a strong reduction as the knockdown of Vps25 (Fig. 6c, d), arguing against the separate function of ESCRT-II in the control of dendritic arbor stability. Altogether, these findings suggest that all three ESCRTs are needed for dendritic arbor stability.

## Discussion

The present study provided evidence of the contribution of ESCRT proteins to dendritogenesis and dendrite maintenance in developing and mature hippocampal neurons, respectively. The link between selected ESCRT proteins and dendritic arbor morphology was previously established mainly in *Drosophila*. The requirement for ESCRT-I, ESCRT-III, and Vps4 for mature neuronal dendrite pruning has been well described [6, 7, 12]. In contrast, information about the contribution of ESCRT modules to dendrite development and stabilization in mammals is quite fragmentary. Our data showed that developing hippocampal neurons that were cultured in vitro, upon the knockdown of all of the tested ESCRT proteins and Vps4, failed to acquire complex dendritic morphology. A similar observation was reported by Lee et al. [13], who knocked down Snf7 in DIV3 cortical neurons. Notably, however, this result was mentioned only as an unpublished observation. Another observation that corroborates the contribution of ESCRT-III to dendritic arbor development in mammals comes from research on hippocampal neurons that were derived from *Alix*^−/−^ knockout mice, in which the complexity of dendritic arbors of DIV13 neurons was significantly lower than in controls [14]. Alix recruits ESCRT-III to the membrane, and this observation indirectly confirms the importance of ESCRT-III for dendritogenesis. Moreover, the requirement of selected ESCRT proteins for mTORC1 activation by insulin in developing neurons and PI3K-stimulated, mTOR-dependent dendritic growth (Figs. 3 and 4) also supports the conclusion that these complexes are needed for dendritic growth in mammalian cells. Our previous studies showed that both mTOR complexes are needed for the growth but not stability of dendritic trees. The knockdown of both mTOR complexes strongly inhibited the positive effects of insulin and PI3K overexpression on the complexity of dendritic arbors of developing hippocampal neurons [24]. Similar results were obtained for the brain-derived neurotrophic factor-induced dendritogenesis of differentiating neural stem cells that were derived from the subventricular zone in postnatal rats [26]. Thus, we postulate that all three ESCRT complexes in developing hippocampal neurons are involved in controlling dendritic growth. However, based on our studies, we cannot exclude their possible contribution to the stabilization of newly formed dendritic branches. Nonetheless, our data clearly showed that ESCRTs in mature neurons are needed for the maintenance of existing dendrites, unlike in *Drosophila* in which their activity is required for dendrite pruning [6]. This finding is in good agreement with the recently published observation that the knockdown of CHMP2B (ESCRT-III) immediately before the full stabilization of dendritic arbors of cultured hippocampal neurons resulted in their simplification [16].

Unknown is the way in which ESCRT proteins contribute to dendrite growth and stabilization at the molecular level. The canonical function of ESCRTs involves the sorting of cellular cargo for degradation. The inhibition of this process results in aberrant trafficking or signaling from internalized receptors. Indeed, we showed that the knockdown of ESCRT-I, ESCRT-II, and ESCRT-III resulted in the accumulation of monoubiquitinylated and polyubiquitinylated conjugates (FK antibody immunofluorescence staining), which is a hallmark of MVB dysfunction [28]. One tempting speculation is that the lack of ESCRT proteins results in a decrease in the degradation of molecules that inhibit dendritic growth, which need to be kept at low levels to avoid the premature maturation of neurons. Alternatively, the lack of ESCRT modules may prevent the degradation of inhibitors of proteins that promote dendrite growth or stability. One example is Deptor, a natural inhibitor of mTORC1 and mTORC2. Verma and Marchese [29] showed that targeting selected ESCRT proteins with siRNA prevented lysosomal Deptor degradation, which in turn rapidly attenuated mTORC2 activation in response to chemokines. We found that the knockdown of selected ESCRT proteins led to mTORC1 downregulation. mTORC1 is an important regulator of dendritic arbor growth [24], and selected ESCRT protein knockdown inhibited dendritic growth that was stimulated by the trophic factor-PI3K signaling pathway. mTOR inhibition alone explains only the retardation of PI3K-induced dendritic growth upon ESCRT inhibition. We did not observe a profound effect of selected ESCRT protein knockdown on mTOR activity in non-stimulated cells, thus excluding the possibility that ESCRTs require mTOR for their actions that are related to basal dendritogenesis. Additionally, the impact of ESCRT protein knockdown on the stability of mature dendrites cannot be explained by mTOR inhibition because this kinase does not appear to be critical for dendritic stabilization [24]. In addition to lysosomal protein degradation, ESCRT modules are required for the maturation of late endosomes and consequently the final steps of autophagy (i.e., the formation of amphisomes by the fusion of autophagosomes with late endosomes or lysosomes) [30]. In neurons, these membranous compartments contribute to protein degradation and signal transduction from axon terminals to the cell body [31]. In fact, the proper maturation and subsequent microtubular transport of axonal endosomes and autophagosomes have profound effects on both dendritogenesis and neuronal survival [31]. Disturbances of autophagic flux were suggested to be a primary cause of disruptions of dendritic arborization and the subsequent neurodegeneration of mature neurons that lack Snf7 [13]. Another potential explanation for the effects of ESCRT knockdown on dendritic arbor maturation and stability comes from studies by the Goldberg group, who observed less-mature mushroom-type dendritic spines in maturating hippocampal neurons with lower ESCRT-III [15, 16]. These authors also reported an increase in thin spines. Mushroom spines are more mature than thin spines and likely pass more current. Indeed, synapses of neurons with diminished CHMP2B have lower efficacy [16]. Neuronal activity and dendritic growth and stability are intimately related [1, 2]. Therefore, one could speculate that insufficient neuronal excitation retards either dendritic growth or stability or both. However, the way in which ESCRT-III or potentially other ESCRTs affect the maturation of dendritic spines is not yet known. The knockdown of some proteins can affect specifically mushroom spines without detrimental effects on dendritic arbors (e.g., Elmo-1 [32]).

ESCRT proteins as a whole complex, separate ESCRT modules, and even individual proteins could contribute to dendrite growth and stability in a non-canonical way. For example, Hrs protein is required for one of the recycling routes of TrkB, a protein that is important for dendritic growth [33]. Another example comes from the study by Konopacki et al. [11], who showed that ESCRT-II in growing axons of *Xenopus* retinal ganglion neurons is exclusively needed for the trafficking and sustained expression levels of DCC, a receptor for the main axon guidance molecule Netrin-1. The authors suggested that ESCRT-II in axons might also be involved in the regulation of mRNA transport and local protein synthesis, similar to *Drosophila* oocytes where ESCRT-II is needed for polarized distribution of the mRNA binding protein Staufen and its target *bicoid* mRNA [10]. Local protein synthesis and Staufen proteins are also important for dendritic growth [22, 34], and similar mechanisms may also operate in dendrites. Such a mechanism could explain very strong effects of Vps25 shRNA compared with shRNAs that target ESCRT-I and ESCRT-III in mature neurons, assuming that equal knockdown efficiency is proven at the protein level. In fact, the knockdown of Staufen 1 in mature neurons leads to strong dendritic arbor simplification, similar to the knockdown of Vps25 (MF and JJ, unpublished observations). However, Vps25 knockdown did not significantly affect the distribution of fluorescently tagged Staufen 1 in primary dendrites (MF and JJ, unpublished observations). Additionally, the transfection of shRNAs that targeted other ESCRT-II components did not have as strong an effect on dendritic arbor stability as Vps25 shRNA. This is important because non-canonical ESCRT-II function in mRNA transport always appeared to require the whole complex. However, we cannot exclude the possibility that the strongest effect of Vps25 shRNA resulted from (i) potentially lower Vps25 protein stability than Vps22 or Vps36 or (ii) stoichiometry of the ESCRT-II complex. It consists of two Vps25 molecules and one each of Vps22 and Vps36. Thus, assuming similar knockdown levels by our shRNAs (at least proven at the mRNA level) for all three proteins, Vps25 knockdown may lead to the faster depletion of ESCRT-II building blocks and significantly lower module formation. Altogether, our study provides the first comprehensive comparison of the contribution of individual ESCRT proteins to dendritogenesis in mammals.

## Electronic Supplementary Material

ESM 1 (PDF 241 kb)
