# Supplementary material for: ESCRT Proteins Control the Dendritic Morphology of Developing and Mature Hippocampal Neurons
Source: Mol Neurobiol. 2018 Nov 7;56(7):4866–79. doi: 10.1007/s12035-018-1418-9 (PMC6647414; doi:10.1007/s12035-018-1418-9)
Supplement: Supplementary file 1 — (PDF 241 kb) [file 12035_2018_1418_MOESM1_ESM.pdf]

Firkowska et al., Online resource 1, Supplementary Figures

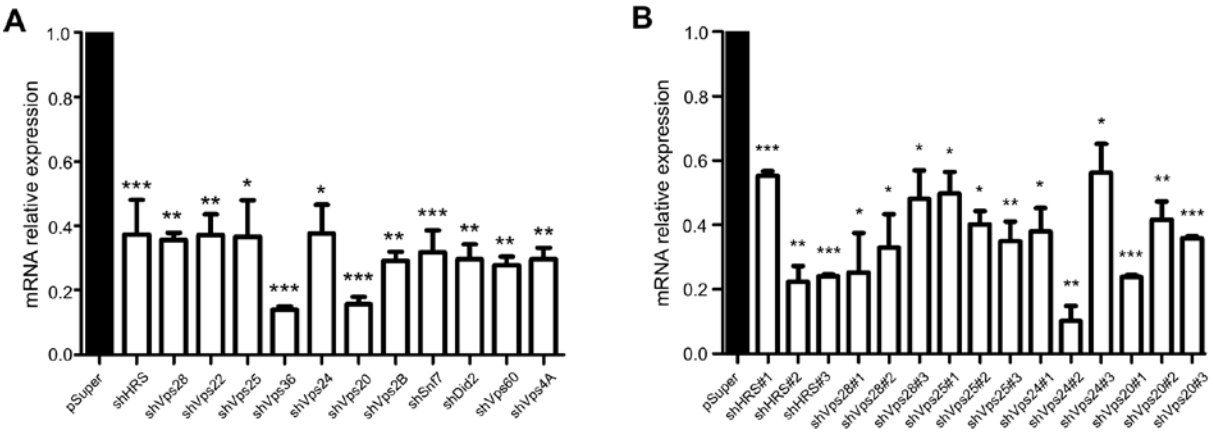

Supplementary Fig. S1. qRT-PCR analysis of shRNA effects. ***A***. The results of the qRT-PCR-based analysis of the indicated gene expression in cortical neurons that were nucleofected on DIV0 with corresponding shRNA pools ***B***. The results of the qRT-PCR-based analysis of the indicated gene expression in cortical neurons that were nucleofected on DIV0 individual shRNAs against HRS (#1, #2, #3), Vps28 (#1, #2, #3), Vps25 (#1, #2, #3) and Vps20 (#1, #2, #3) (Table 1) for 3 days. GAPDH mRNA was used as a reference. The plots represent 2(−ΔΔCt) mean values ± SEM. Data are derived from three independent experiments. $*p < 0.05$, $**p < 0.01$, $***p < 0.001$ (one-sample *t*-test).

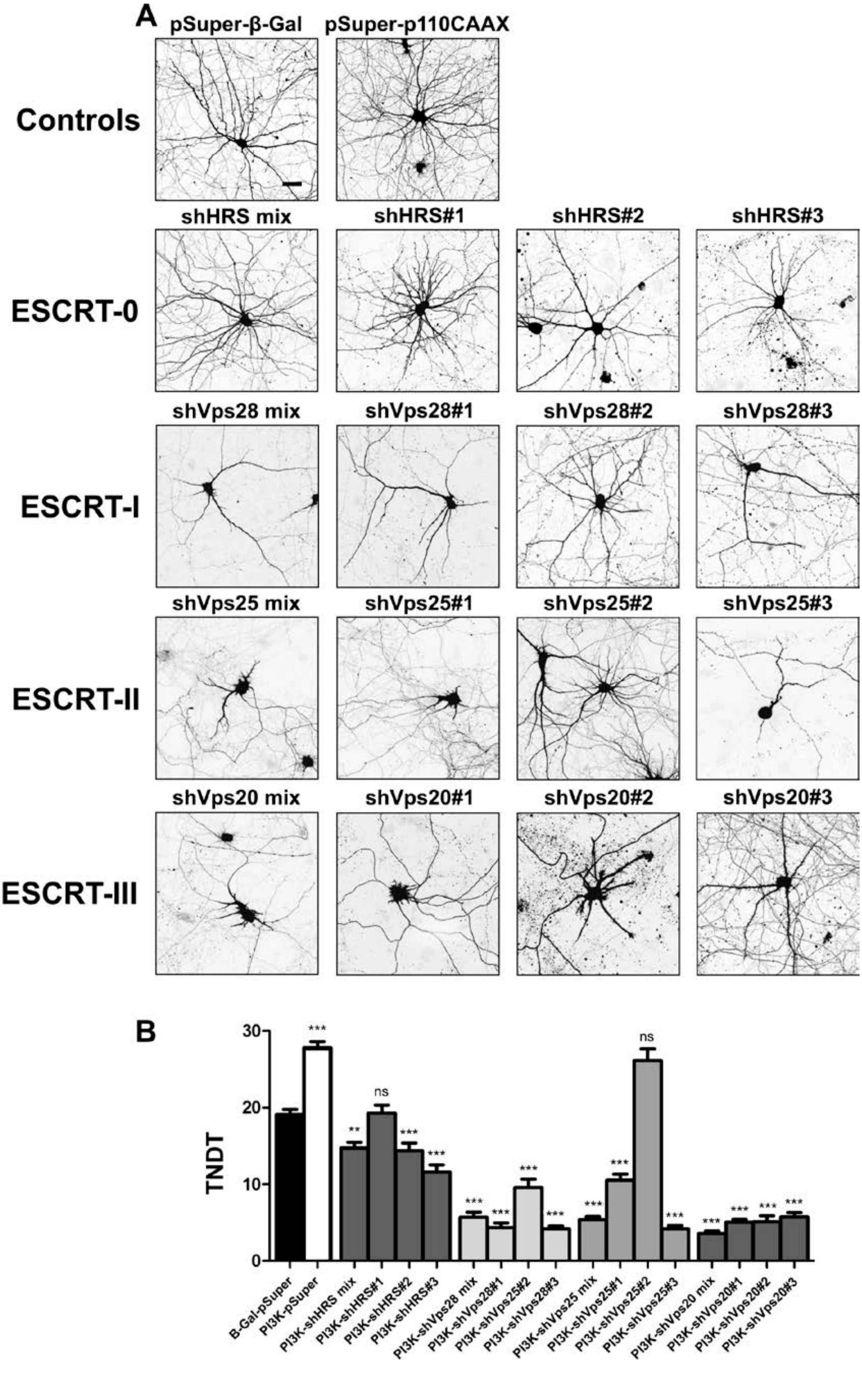
A
pSuper-β-Gal
pSuper-p110CAAX
Controls
shHRS mix
shHRS#1
shHRS#2
shHRS#3
ESCRT-0
shVps28 mix
shVps28#1
shVps28#2
shVps28#3
ESCRT-I
shVps25 mix
shVps25#1
shVps25#2
shVps25#3
ESCRT-II
shVps20 mix
shVps20#1
shVps20#2
shVps20#3
ESCRT-III
B
TNDT
30
20
10
0
***
ns
**
***
***
ns
B-Gal-pSuper
PI3K-pSuper
PI3K-shHRS mix
PI3K-shHRS#1
PI3K-shHRS#2
PI3K-shHRS#3
PI3K-shVps28 mix
PI3K-shVps28#1
PI3K-shVps25#2
PI3K-shVps28#3
PI3K-shVps25 mix
PI3K-shVps25#1
PI3K-shVps25#2
PI3K-shVps25#3
PI3K-shVps20 mix
PI3K-shVps20#1
PI3K-shVps20#2
PI3K-shVps20#3

Supplementary Fig. S2. Comparison of phenotypic effects of pools and individual shRNAs that were used to study PI3K-stimulated dendritogenesis. *A.* Representative images of neurons that were transfected on DIV7 for 5 days with p110-CAAX plasmid and pSuper, a pool of three shRNAs, or individual shRNAs against HRS, Vps28, Vps25, or shVps20. For the visualization of neuronal morphology, the cells were co-transfected with a GFP-encoding plasmid. Scale bar = 40 µm. ***B.*** Total number of dendritic tips (TNDT) of cells that were transfected as in *A*. Cell images were obtained from four independent experiments. Number of analyzed cells: pSuper-β-Gal (n = 65), pSuper-p110CAAX (n = 63), shHRS-mix (n = 56), shHRS#1 (n = 57), shHRS#2 (n = 49), shHRS#3 (n = 50), shVps28 mix (n = 46), shVps28#1 (n = 46), shVps28#2 (n = 44), shVps28#3 (n = 53), shVps25 mix (n = 47), shVps25#1 (n = 46), shVps25#2 (n = 57), shVps25#3 (n = 42), shVps20 mix (n = 60), shVps20#1 (n = 61), shVps20#2 (n = 55), shVps20#3 (n = 58). Error bars indicate the SEM. ***$p < 0.001$, compared with pSuper/p110-CAAX (Kruskal-Wallis test followed by Dunn's *post hoc* test); ns, not significant.
